# Comparison between indicine and taurine cattle DNA methylation reveals epigenetic variation associated to differences in morphological adaptive traits

**DOI:** 10.1080/15592294.2022.2163363

**Published:** 2023-01-04

**Authors:** E. Capra, B. Lazzari, M. Milanesi, G. P. Nogueira, J. f. Garcia, Y.T. Utsunomiya, P. Ajmone-Marsan, A. Stella

**Affiliations:** a Institute of Agricultural Biology and Biotechnology, National Research Council IBBA CNR, Lodi, Italy; b School of Veterinary Medicine, Araçatuba, Department of Production and Animal Health, São Paulo State University (Unesp), Araçatuba, Brazil; c International Atomic Energy Agency, Collaborating Centre on Animal Genomics and Bioinformatics, Araçatuba, Brazil; d Department for Innovation in Biological, Agro-Food and Forest Systems (DIBAF), University of Tuscia, Viterbo, Italy; e Department of Animal Science, Food and Nutrition – DIANA, and Nutrigenomics and Proteomics Research Center – PRONUTRIGEN, Università Cattolica del Sacro Cuore, Piacenza, Italy

**Keywords:** Methylation, bovine, evolution, adaptation, breed, cattle, epigenetic, species

## Abstract

Indicine and taurine subspecies present distinct morphological traits as a consequence of environmental adaptation and artificial selection. Although the two subspecies have been characterized and compared at genome-wide level and at specific loci, their epigenetic diversity has not yet been explored. In this work, Reduced Representation Bisulphite Sequencing (RRBS) profiling of the taurine Angus (A) and indicine Nellore (N) cattle breeds was applied to identify methylation differences between the two subspecies. Genotyping by sequencing (GBS) of the same animals was performed to detect single nucleotide polymorphisms (SNPs) at cytosines in CpG dinucleotides and remove them from the differential methylation analysis. A total of 660,845 methylated cytosines were identified within the CpG context (CpGs) across the 10 animals sequenced (5 N and 5 A). A total of 25,765 of these were differentially methylated (DMCs). Most DMCs clustered in CpG stretches nearby genes involved in cellular and anatomical structure morphogenesis. Also, sequences flanking DMC were enriched in SNPs compared to all other CpGs, either methylated or unmethylated in the two subspecies. Our data suggest a contribution of epigenetics to the regulation and divergence of anatomical morphogenesis in the two subspecies relevant for cattle evolution and sub-species differentiation and adaptation.

## Introduction

Modern day cattle belong either to the taurine or the indicine sub-species, which derive from independent domestication events. European taurine breeds are characterized by excellent carcass and meat quality or high milk production potential, but are poorly adapted to harsh environments [1,2]. Indicine breeds are more adapted to tropical wet/dry semi-arid, arid and hot environments and to parasites, and possess distinct morphological traits such as a hump, large ears, and excess skin [3,4].

Although the two cattle subspecies have been deeply characterized for genetic differences at the genome-wide level and at specific loci, phenotypic differences between them can only partially be explained by genomic variants [5–8]. The non-genetic proportion of phenotypic variation has been defined as phenotypic plasticity and can in part be attributed to epigenetic variation, standing at a cross-road between genetic and environmental variance [9,10]. This effect was largely described in asexually reproducing invertebrates and in some vertebrates [11]. Epigenetic variation was also proposed as a mechanism triggered by animal domestication able to shape phenotypic features of domesticated animals in very short time scales [12]. Literature data on dogs and grey wolves reported specific differences in methylation patterns between the two species, suggesting that epigenetic mechanisms might play an important role in early steps of domestication [13]. Epigenetic variation has a higher plasticity than genetic variation and can cope better with environmental fluctuations [14]. However, the contribution of epigenetic variation in explaining the missing heritability of phenotypic traits is not well understood, because transgenerational transmission, persistence over time and stabilization within the livestock genome are still to be explored [14].

In farming, behaviour, diet, stress, and environmental variation have a strong impact on the animal epigenome [15,16]. In pigs and sheep, the impact has been reported to persist along multiple generations [17,18]. Recently, we reported genome-wide DNA methylation changes in blood samples from indicine (Nellore) and taurine (Angus) breeds under heat stress [19]. After a stressful period, Nellore showed methylation changes in genes related to cellular defence and stress response, whereas Angus (A) response was less focused. The overall methylation profiles in Nellore (N) and A animals showed remarkable diversity between the two subspecies that was independent of the environmental challenge and presumably related to their origin, breed characteristics, and polymorphisms at CpG islands [19]. In pigs, epigenome-wide muscle profiling has been reported to show important differences across breeds, probably as a result of long-term selection for quantitative traits, involving a very high number of genes [20]. While in the previous investigation [19], differential methylated regions (DMRs) were identified comparing individual breeds across environmental conditions, here we use Reduced Representation Bisulphite Sequencing (RRBS) data from N and A animals, to identify cytosines and regions differentially methylated between breeds and to investigate the potential role played by epigenetics in subspecies domestication. The same animals have also been fully sequenced to distinguish differential methylation signals from polymorphisms at CpG dinucleotides, to focus on epigenetic differences while getting rid of the bias generated by genetic differences existing between the two subspecies.

## Materials and methods

### Animal sampling

A total of 5 N and 5 A healthy young bulls of about 15 months of age were investigated. Half-sib animals within each breed were selected to minimize genetic variation. A and N bullocks were purchased at 7 months of age. Angus were from Uruguaiana (Rio Grande do Sul state, Brazil), where this breed has been present for more than 100 years and the climate is temperate, humid, with hot summers (Cfa) according to the Köppen Geiger classification [21,22]; Nellore was from Dourados (Mato Grosso do Sul state, Brazil, tropical zone) where climate is tropical with dry winter (Aw). A and N animals were transported to the experimental station (located at −21.186244 latitude and −50.439053 longitude) at UNESP Aracatuba (Sao Paulo state, Brazil, tropical zone) where climate is also classified Aw. During the adaptation period in Araçatuba, animals were kept in two 200-square-metre paddocks, 100 square metres of which were covered by a shading net (80% sunblock), with regular access to pasture (60 days). Animal groups were thereafter kept without shade for 56 days (challenge period). During the recovery period, the shading nets were replaced such that all animals were kept with shade available and were allowed access to pasture until slaughter (30 days). Sampling was performed at the peak heat stress period, and at the end of recovery period, during the cool season, after full recovery from heat stress. (C = stressful challenge and R = Recovery period) [19].

### DNA isolation

DNA was isolated from whole blood with QIAamp DNA Blood Midi Kits (Qiagen) following manufacturer instructions.

### Reduced representation bisulphite sequencing RRBS

One μg of genomic DNA was used for RRBS libraries preparation as previously reported by Del Corvo et al., 2021 [19]. RRBS data are available at the Sequence Reads Archive (SRA), BioProject accession number, PRJNA675605.

### Genotyping by sequencing (GBS)

Genotyping by sequencing (GBS) was performed using different approaches. In order to increase the CpG coverage in cytosine selected for RRBS, each sample was processed following two different library preparations, with or without a pre-treatment with MspI.

MspI pre-treated libraries were obtained after DNA digestion (200 ng) with MspI (New England Biolabs, Ipswich, MA, United States) by overnight incubation at 37°C, following the manufacturer instructions. After purification with ampure beads (Vol 1:1), libraries were generated using TruSeq Nano DNA Library Preparation Kit (Illumina, San Diego, CA, United States), without a Covaris DNA fragmentation step. Standard GBS libraries were also prepared using the TruSeq Nano DNA Library Preparation Kit, following manufacturer instructions. Libraries were sequenced on an Illumina Hiseq X (San Diego, CA, United States) to generate 150-base paired-end reads. GBS Data are available at the Sequence Reads Archive (SRA), BioProject accession number, PRJNA855305.

### SNP detection

GBS reads were quality controlled with FastQC v0.11.9 (http://www.bioinformatics.babraham.ac.uk/projects/fastqc/). Sequences were then trimmed with TrimGalore v0.6.4 (http://www.bioinformatics.babraham.ac.uk/projects/trim_galore/) imposing a cut-off at sequence quality score below 20. The resulting average sequence quality was between 37 and 39. Sequences were mapped to the *Bos taurus* reference genome (ARS-UCD1.2: GCF_002263795.1) with BWA-MEM v0.7.17 [23]. SNPs were detected with Freebayes v1.3.5 [24].

### Methylation analysis

Illumina sequence reads were analysed using nf-core [25], through the nf-core/methylseq v1.5 pipeline (doi: 10.5281/zenodo.2555454) selecting Bismark v0.22.3 (https://www.bioinformatics.babraham.ac.uk/projects/bismark/) as the aligner. The pipeline includes FastQC v0.11.9. (http://www.bioinformatics.babraham.ac.uk/projects/fastqc/) for raw data quality control, Trim Galore v0.6.4 (http://www.bioinformatics.babraham.ac.uk/projects/trim_galore/) for adapter sequence trimming, and Qualimap v2.2.2 for alignment quality control [26].

Sequence reads from all samples were aligned to the bisulphite-converted *Bos taurus* reference genome (ARS-UCD1.2: GCF_002263795.1), and methylation calls were extracted using the Bismark methylation extractor v0.22.3 function. The Seqmonk software v1.47.1 was used for visualization and analysis of the Bismark output. In order to identify cytosines suitable for differential methylation analysis and get rid of variant positions between A and N genomes, cytosines matching SNP positions in either species were identified by in-house developed scripts and removed from the analysis. The overall cytosine methylation distribution in all the 20 samples (5 N and 5 A animals in the C and R periods) was assessed by Principal Component Analysis (PCA) considering only cytosines with at least 10X coverage in all samples. A second PCA analysis was performed on the ten animals (5 A and 5 N), after grouping samples from the C and R periods. Variance partition analysis was calculated with the ‘variancePartition’ R package (http://bioconductor.org/packages/release/bioc/html/variancePartition.html). Differentially methylated cytosines (DMCs) between the two sub-species were detected among cytosines with at least 10X coverage in all ten animals, using the Edge-R statistical package (Bioconductor, https://bioconductor.org/packages/release/bioc/html/edgeR.html). Differential methylation was assessed using two filters: False Discovery Rate (FDR) ≤ 0.05, and absolute cut-off of 10% (i.e., at least 10% methylation difference between the two subsets). Visualization of CpG methylation level was performed using the Methylation plotter Software [27].

### Gene ontology analysis

Genes encompassing DMCs were ranked based on DMC frequency in the gene, normalized according to gene length (n° of DMCs/gene length). Gene ontology (GO) classification was performed only on genes bearing two or more close DMCs (<2000 bps of distance between cytosines), using the Cytoscape plug-in ClueGO, which integrates GO [28] and enhances biological interpretation of large lists of genes.

## Results

### Global mapping of DNA methylation and PCA analysis

The RRBS data used in this study derive from a previous investigation which explored the CpG methylation variation in Nellore and Angus steers in response to heat stress [19]. Differential methylation analysis was conducted within breed to evaluate breed-specific response in the two subspecies.

In the present study we focus on CpG methylation variation between subspecies. In addition, we add to previous RRBS data GBS data from each animal. The GBS data allowed us to correctly compute differences in methylation levels between indicine and taurine subspecies by disentangling methylation from genomic variation [29]. In fact, bisulphite treatment causes the conversion of unmethylated cytosines into uracil, and into thymine during the following PCR reactions. Thymines detected in the bisulphite sequencing experiments are therefore interpreted as unmethylated cytosines. For this reason, a distinction between thymines resulting from the deamination of unmethylated cytosines and real thymines, deriving from mutation, was necessary, to clarlfy results from this possible bias.

To evaluate the epigenetic variation in the two sub-species, RRBS reads were mapped to the *Bos taurus* genome (ARS-UCD1.2). A similar efficiency was observed in mapping *taurus* and *indicus* sequences (44.8% for A and 44.4% for N), indicating that the choice of the reference genome had little influence on cytosine methylation detection. In RRBS analysis, the cut-off of 10X coverage for all ten A and N animals was imposed. The average number of reads per sample was 14.8 M (range: 9.2 M–25.2 M). About 74.1% and 73.4% of the CpG-enriched regions represented in RRBS were methylated in A and N, respectively (Supplementary file 1).

All animals participating to the study were whole-genome sequenced. Sequences were mapped to the *Bos taurus* genome and genomic positions of SNPs recorded. Cytosines corresponding to SNPs between the reference genome and the sequenced animals (referred to as c-SNPs) were removed from the methylation analysis, to avoid data misinterpretation due to the erroneous attribution of a SNP to an unmethylated cytosine. A total of 34,677 cytosines (4.97% of the full cytosine dataset) were discarded because matching with SNPs in one or both subspecies. Further 1,788 cytosines were discarded as positioned ± 1 bp from identified SNPs, as these SNPs have an effect on the CpG dinucleotide, which is modified into either CpT, CpC, or CpA, which cannot be attributed to the CpG context. The resulting c-SNP-free dataset comprised 660,845 cytosines within the CpG context. All the subsequent analyses were run on this final dataet, which represents the positions where differential methylation can be trustfully investigated.

**Figure 1.**
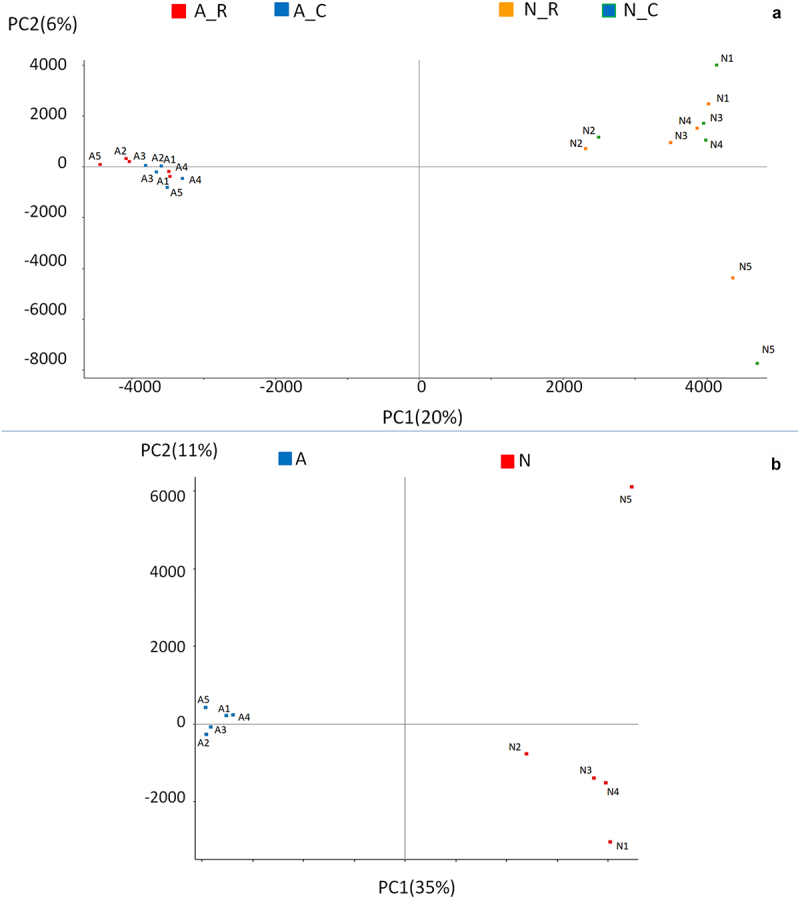
Principal Component Analysis of total CpGs in Angus and Nellore animals considering: a) individuals in the two seasons (20 samples) and b) individuals grouped across the hot (C = Challenge) and cool seasons (R = Recovery) (10 samples).

Principal component analysis of the total CpGs in A and Nanimals in the C = stressful Challenge and R = Recovery periods shows that the overall RRBS profiling discriminates well A and N sub-species. As previously shown, different environmental conditions such as heat-stress affect the differential methylation of a limited number of specific CpG sites [19], but have very little or no influence on the overall methylation genomic distribution (Figure 1a). Variance partition analysis showed that individuals, breed and season (C and R), contributed for 13.15%, 3.48%, and 1.24% of total variance, respectively. The complement to 100% was all residual variance. Following this observation, PCA analysis was performed on merged (C and R) sequences and confirmed a high level of methylation diversity between subspecies, as well as a greater homogeneity among A animals with respect to N (Figure 1b). One of the N animals was an outlier on PC2.

### Differentially methylated cytosines (DMCs) analysis

**Figure 2.**
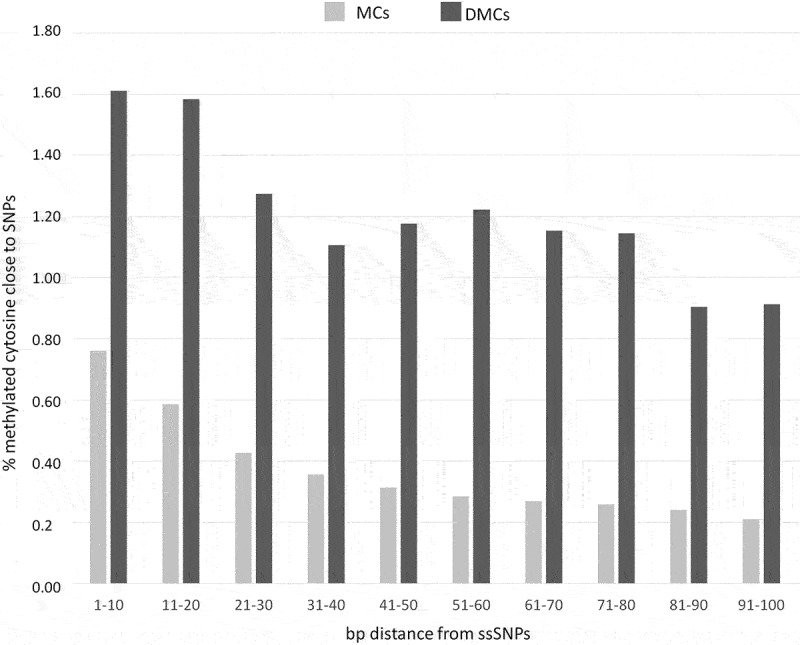
Proportion of methylated cytosines in DMCs and MCs subsets flanking subspecies-specific SNPs.

PCA analysis showed a high diversity between the two subspecies. Using the high threshold used here the comparison of A and N subspecies in the stressed and recovery seasons revealed no DMC, indicating that between breed comparison identified signals not influenced by contingent environmental conditions. On the contrary, when between sub-species CpG methylation diversity was considered, a total of 25,765 DMCs (3.90% of the total cytosines c-SNP-free dataset) were identified between A and N (Supplementary file 2). The GBS analysis identified a total of 1,701,571 positions showing the two subspecies to be fixed for alternative bases. To investigate possible relationships between subspecies-specific SNP (ssSNP) distribution and methylation, we compared SNP positions recovered from the resequencing analysis and the positions of methylated cytosines relative to the closest SNP. We considered DMCs (25,765 in total) as well as methylated cytosines not exhibiting differential methylation between A and N (referred to as MCs, 635,080 in total). The proportion of DMCs and MCs near ssSNPs was counted within ten bp intervals moving away from ssSNP: 1–10 bps,11–20 bps, 21–30 bps, 31–40 bps, 41–50 bps, 51–60 bps, 61–70 bps, 71–80 bps, 81–90 bps, and 91–100 bps (Figure 2).

A higher proportion of cytosines close to SNPs can be observed in DMCs with respect to MCs at each of the considered intervals. Interestingly, some differences can be highlighted in MCs and DMCs profiles: while MCs exhibit a progressive decrease when moving away from ssSNPs, DMC frequencies show a less progressive and less consistent decrease.

### DMCs annotation and functional enrichment GO-analysis

**Figure 3.**
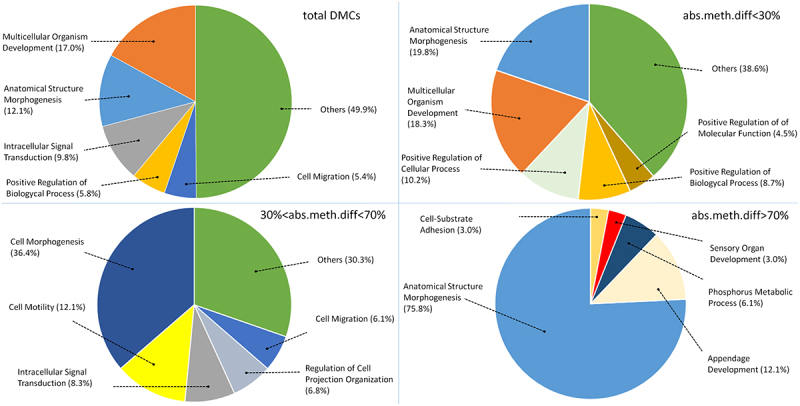
Gene Ontology analysis on total DMCs, DMCs below 30% abs.meth.diff, DMCs between 30% and 70% abs.meth.diff and DMCs above 70% absolute difference in methylation level (abs.meth.diff.).

In order to perform Gene Ontology (GO) analysis, four different sets of DMCs were considered. GO analysis was first performed on total DMCs, that included all CpG methylation variations between A and N animals. The three additional subsets were based on the absolute difference in methylation level (abs.meth.diff), defined as the percentage of differential methylation observed in DMCs (i.e., the % of cytosines exhibiting differential methylation between the two subspecies in each DMC), and three classes were defined, of <30%, 30–70%, and >70%. In total 5,742 differentially methylated genes (DMGs) were identified by GO analysis. Some genes had regions falling within two different classes, resulting in the three abs.meth.diff groups including 4,046, 2,909, and 1,000 DMGs, respectively. Functional analysis was performed on the four retrieved gene sets and, interestingly, all of them showed enrichments in genes related to anatomical structure morphogenesis, cellular morphogenesis and multicellular organism development. Furthermore, GO analysis of the subset with the highest difference in methylation (>70%) identified genes with functions that were prevalently associated with anatomical structure morphogenesis (over 75%), (Figure 3).

**Figure 4.**
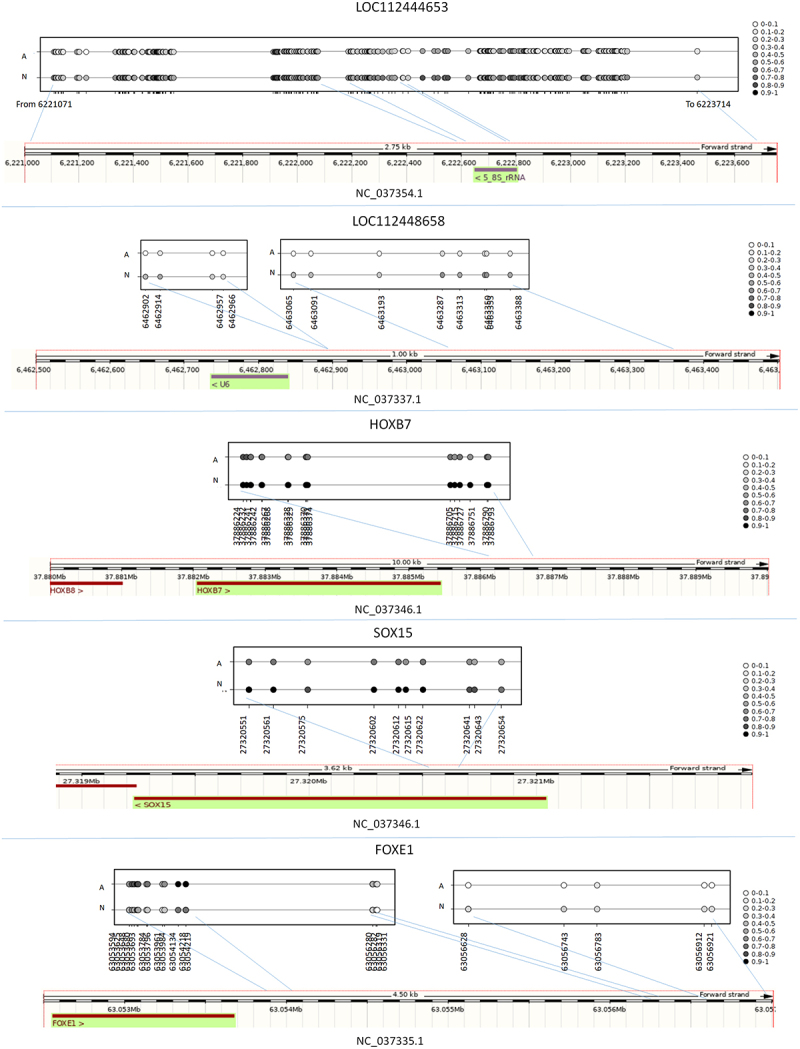
Representation of average level of CpG methylation in A and N animals for different genes presenting a high number of close differentially methylated stratches: LOC112444653, LOC112448658, HOXB7, SOX1, FOXE1.

To identify genes likely to be selectively targeted by differential methylation, we explored CpG distribution by retrieving CpGs within 1Kb from each other. Nearby genes were then assigned to groups based on the presence of clusters of two or more DMCs with similar methylation status. Out of a total of 5,742 DMGs, 3,265 had no nearby DMC clusters, 1,877 (32.7%) showed at least two closely located DMCs, 921 (16.0%) at least three sets and 600 (10.4%) more then three close DMCs. GO analysis of the latter three subsets identified pathways associated to anatomical structure morphogenesis, system development, and cell differentiation (Supplementary file 3). Interestingly, several genes associated to anatomical structure morphogenesis presented long (more then three) CpG stretches (Supplementary file 3 and Figure 4).

## Discussion

In this study, a first characterization of the single cytosine methylation variation between *taurine* and *indicine* cattle subspecies was obtained by RRBS. Aand N RRBS profiling from blood samples showed an average level of CpG methylation of about 74%. This result was not consistent with previous data obtained by RRBS profiling of blood from tropical bovine breeds, which ranged from 51% to 57% [30]. A possible cause of this inconsistency lies in the different protocol used, in particular in size selection for library preparation [31]. To increase genome coverage, in our analysis, we selected a broad range of MspI digests including large fragments, enriched in non-dense, and promoter poor, CpG regions. The study from Sevane et al. identified 334 differentially methylated regions (each containing 4 or more CpGs) on 20,234 detected MCs (1.65%) between Colombian Creole cattle breeds and their putative Spanish ancestors. In our study, about 4% of the total CpGs detected showed variation between A and N animals (25,765 DMCs). As expected, the proportion of the epigenome that differs between subspecies is much greater than between animals from the same subspecies.

Recently, Costes et al., 2022, explored the cattle sperm CpG methylation diversity in 120 French Montbéliarde bulls. Differential CpG methylation calling was profoundly affected by sequence polymorphism [32]. This hampered the distinction between both genetic and epigenetic variations (C/T variations called as unmethylated cytosines). In their study, all putative variants recorded in the 1000 Bull Genomes database [32] were filtered out from the CpGs identified by RRBS.

As the two breeds here analysed belong to different subspecies and are known to be genetically distant, we included in our approach a strategy to eliminate any confounding factor deriving from the incorrect attribution of polymorphisms in CpG dinucleotides to methylation differences, using GBS data obtained by sequencing the whole genome of all animals analysed. Cytosines immediately adjacent to SNPs (± 1 bp) were also excluded to maintain the analysis restricted to CpG context, also avoiding a further bias due to the conversion in non-CpG motifs (CpC, CpT and CpA) that are known to be methylated with low frequency in mammals [33].

Following the robust assessment of the cytosine methylation state and SNP variation in our samples, we investigated the relationship between epigenetic and genetic variation in the two breeds. We found a higher frequency of breed-specific SNPs in DMCs regions, compared to non-DMCs. Correlation between CpG methylation and genetic variation has been previously reported in human populations, and it has been partially attributed to the evolutionary role of DNA methylation as intermediate remodulator of phenotypic differences [34,35]. In these studies, one-third of the DNA methylation differenceswere not related to genetic variation, suggesting the existence of an independent contribution of epigenetic variability to natural human phenotipic variation [34,35]. Our results identified a high number of CpGs and several genes showing differential methylation above 70% between A and N animals. These high values are likely indicating differential epimutation of both alleles in the two breeds. In our study, di-allelic epimutations prevalently occurred in genes related to anatomical morphogenesis that impact animal phenotype during adaptation. Whether epigenetic modification can be transmitted to subsequent generations remains a debated topic [36,37]. Recently, in Drosophila it was shown that stress causes specific epigenetic modifications that generate phenocopies with the corresponding loci more susceptible to DNA alterations making phenotypic variants more heritable [38]. Several studies reported that epigenetic mechanisms can produce heritable phenotypes in animals, such as body size variation in ants [39], reproductive seasonality in great tits [40], salinity adaptation in three spined sticklebacks [41] and eye development in cavefish [42]. All these studies reported a higher frequency of methylation variation on cytosines in genomic regions where genes related to phenotype alteration are located. Methylation changes may therefore contribute to promote genetic variation in genomic regions associated to adaptation and therefore may play a role in evolution.

Interestingly, the A and N subspecies comparison identified many cytosines up or down methylated in A and N, in many cases grouped in long CpG stretches in specific genomic regions, including many genes and LOC genes. Several genes having a high number of differentially methylated CpGs in relation to gene size code for transcription factors that regulate organ and tissue morphogenesis. In particular, HOXB7 is a regulator of proliferation of mesenchymal progenitors and osteogenesis [43], FOXE1 displays its function in hair follicle morphogenesis [44], SOX15 has an essential role in regionalizing stratified squamous epithelium [45], and NKX2-3, a member of the NK2 homeobox family of transcription factors, activates the bone formation signalling pathway during tooth morphogenesis [46]. Four out of the five LOC genes presenting the longest differentially methylated CpG stretches in relation to gene size (LOC112444653, LOC112448658, LOC112448208 and LOC104976084) showed methylation variation in the promoter region. Interestingly, LOC112444653, a 5.8S ribosomal RNA gene, showed the longest differentially methylated stretch (about 305 CpGs). LOC112444653 methylation has recently been reported to change in mammary gland tissue between cows producing milk with high and low fat and protein content [47]. This genomic region is involved in tissue formation and animal physiological characteristics. Interestingly, methylation variation at this gene was observed in two different tissues: mammary gland and whole blood. Methylation variation at this locus may influence early stages of tissue morphogenesis and modifications are retained in different cellular types in adult animals. LOC112444653 methylation was recently reported to change in peripheral blood mononuclear cells from Holstein and Jersey cows in response to heat stress. The locus has a potential role in regulating animal adaptive plasticity as a function of environmental conditions [48].

## Conclusion

Our results suggest that the RRBS profiling in different breeds and/or subspecies provide an insight on the molecular basis of epigenetic regulation of cattle adaptation. RRBS profiling of A and N animals shows a high epigenetic diversity between breeds belonging to different subspecies, not influenced by external environmental conditions, but more likely related to evolutionary trajectories. Our results support the assumption that epigenetic diversity between the two subspecies can also be influenced by genetic polymorphism and can reflect the genomic evolutionary history between *Bos indicus* and *Bos taurus* cattle, but also support the hypothesis that epigenetic differences between A and N animals represent a source of variation with an impact on anatomical morphogenesis. On the other hand, how these epigenetic signatures are retained during zygotic demethylation or are re-established in somatic tissues remains to be explored.

## Supplementary Material

Supplemental Material

## Data Availability

All NGS sequencing data have been deposited in the Sequence Reads Archive (SRA). The BioProject accession number for RBS and GBS data are PRJNA675605 (https://www.ncbi.nlm.nih.gov/bioproject/PRJNA675605) and PRJNA855305 (https://www.ncbi.nlm.nih.gov/bioproject/PRJNA855305) , respectively.

## List of abbreviations

RRBS: Reduced Representation Bisulfite Sequencing, A: Angus, N: Nellore; GBS: Genotyping by sequencing; SNPs: single nucleotide polymorphisms, CpG: cytosines in CpG dinucleotides, DMCs: differentially methylated cytosines, C: stressful Challenge, R: Recovery, ssSNP: subspecies-specific: single nucleotide polymorphism, MCs: methylated cytosines, GO: Gene Ontology, abs.meth.diff: absolute difference in methylation level, DMGs: differentially methylated genes, PCA: Principal Component Analysis, FDR: False Discovery Rate.
