# Supplementary material for: Comparison between indicine and taurine cattle DNA methylation reveals epigenetic variation associated to differences in morphological adaptive traits
Source: Epigenetics. 2023 Jan 4;18(1):2163363. doi: 10.1080/15592294.2022.2163363 (PMC9980582; doi:10.1080/15592294.2022.2163363)
Supplement: Supplemental Material [file KEPI_A_2163363_SM3983.zip › Supplementary files/Additional file 1.docx]

**Additional file 1.** Mapping and methylation statistics. A=Angus; N=Nellore; C=Challenge; R=Recovery.

| **Sample** | **Mapping %** | **Total Cs** | **CpGs meth** | **CpGs not meth** | **Methylation %** | **Mean %** | **SD %** |
| --- | --- | --- | --- | --- | --- | --- | --- |
| A1_C | 43.0 | 2.71E+08 | 22936201 | 8354660 | 73.3 | 74.1 | 0.93 |
| A1_R | 44.3 | 3.85E+08 | 31736484 | 11383739 | 73.6 |  |  |
| A2_C | 45.2 | 2.75E+08 | 23112566 | 7472972 | 75.6 |  |  |
| A2_R | 46.4 | 3.04E+08 | 24891807 | 8509203 | 74.5 |  |  |
| A3_C | 40.3 | 3.09E+08 | 25311972 | 8655726 | 74.5 |  |  |
| A3_R | 46.0 | 3.51E+08 | 28420620 | 10233846 | 73.5 |  |  |
| A4_C | 46.6 | 3.18E+08 | 26122712 | 8442877 | 75.6 |  |  |
| A4_R | 45.7 | 4.5E+08 | 36141144 | 13506272 | 72.8 |  |  |
| A5_C | 45.1 | 3.05E+08 | 25218653 | 9002866 | 73.7 |  |  |
| A5_R | 45.6 | 3E+08 | 24518583 | 8679181 | 73.9 |  |  |
| N1_C | 44.0 | 2.25E+08 | 18624964 | 7331023 | 71.8 | 73.4 | 0.90 |
| N1_R | 42.2 | 2.76E+08 | 22917615 | 8592611 | 72.7 |  |  |
| N2_C | 44.8 | 3.17E+08 | 25742207 | 9334119 | 73.4 |  |  |
| N2_R | 44.8 | 5.41E+08 | 44264805 | 15546828 | 74.0 |  |  |
| N3_C | 45.8 | 3.08E+08 | 25440027 | 8982612 | 73.9 |  |  |
| N3_R | 43.0 | 4.34E+08 | 35860258 | 13026295 | 73.4 |  |  |
| N4_C | 42.3 | 2.71E+08 | 22324516 | 8335052 | 72.8 |  |  |
| N4_R | 45.7 | 3.77E+08 | 31985936 | 10570562 | 75.2 |  |  |
| N5_C | 42.8 | 1.88E+08 | 15624915 | 5711561 | 73.2 |  |  |
| N5_R | 48.2 | 2.85E+08 | 21337132 | 7823402 | 73.2 |  |  |
