# Supplementary material for: Comparison between indicine and taurine cattle DNA methylation reveals epigenetic variation associated to differences in morphological adaptive traits
Source: Epigenetics. 2023 Jan 4;18(1):2163363. doi: 10.1080/15592294.2022.2163363 (PMC9980582; doi:10.1080/15592294.2022.2163363)
Supplement: Supplemental Material [file KEPI_A_2163363_SM3983.zip › Supplementary files/Additional file 3.docx]

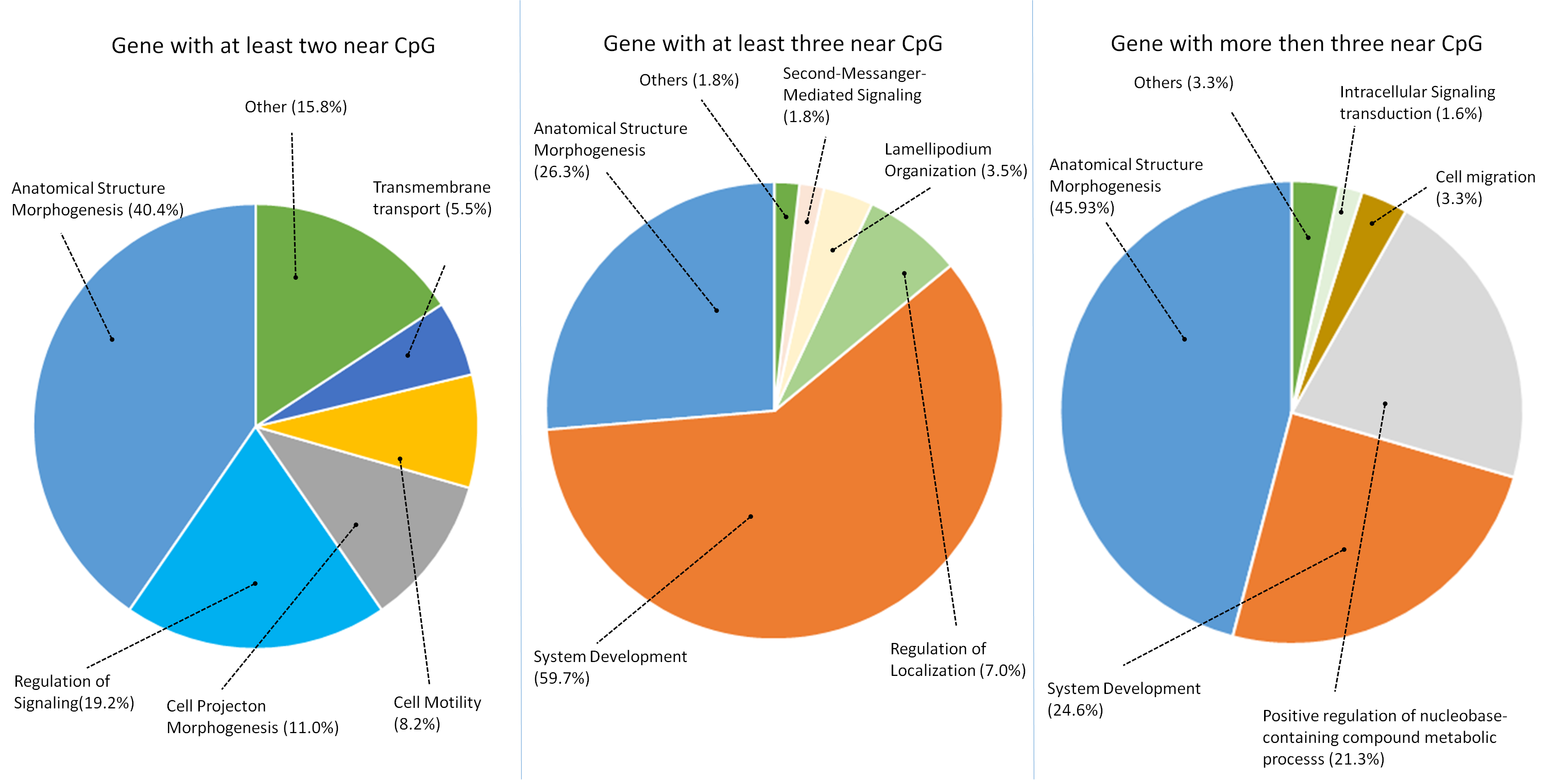


**Additional file 3.** Gene Ontology analysis on DMCs with selected for their proximity: with al least two near DMCs, with al least three near DMCs, with more then three near DMCs.
